# Xenograft tissue slice tandem co-cultures are a highly specific model to selectively analyze drug inhibitory effects on glioblastoma invasion

**DOI:** 10.1016/j.jbc.2025.110986

**Published:** 2025-11-25

**Authors:** Kim K. Wagner, Aileen Heinze, Tamara Zenz, Andrea J. Yool, Heike Franke, Achim Aigner

**Affiliations:** 1 University of Adelaide, School of Biomedicine, Faculty of Health and Medical Sciences, Adelaide, Australia; 2 Universität Leipzig, Rudolf-Boehm-Institute for Pharmacology and Toxicology, Leipzig, Germany; 3 Universität Leipzig, Rudolf-Boehm-Institute for Pharmacology and Toxicology, Clinical Pharmacology, Leipzig, Germany; 4 Comprehensive Cancer Center Central Germany (CCCG), Site Leipzig, Germany

**Keywords:** 3D tumor model, air-liquid interface model, anticancer drug, drug screening, glioblastoma, HDAC inhibitor, invasion, immunohistochemistry, temozolomide, tissue slice culture, vorinostat, entinostat, apamin

## Abstract

Test systems enabling preclinical assessment of drug effects in relevant models are essential for optimizing the selection of candidate therapeutics before their further clinical translation. Xenograft tissue slice tandem co-culture (XTCC) models were developed as *ex vivo* systems for visualizing glioblastoma (GBM) tumor growth and invasion into the complex host tissue structures of the brain. Work here tested the XTCC model for delineating specific drug effects, in particular inhibition of invasion as major issue in GBM. The established chemotherapeutic Temozolomide (TMZ) and three promising candidates – two histone deacetylase inhibitors, Vorinostat and Entinostat, and the neuropeptide Apamin – were tested. XTCCs were generated by placing G55T2 or U87-MG cell-derived tumor xenograft tissue slices onto murine cortical brain slices. Upon drug treatment, effects on growth, invasion, proliferation, and apoptosis were analyzed by immunohistochemistry. Differences in invasion capacity were seen between the two cell lines. Profound invasion-inhibitory effects of 100 μM TMZ were accurately monitored and substantially higher than inhibition of the bulk tumor mass. Likewise, the extent of single-cell invasion into the normal brain tissue was massively inhibited by Vorinostat and especially by Entinostat, indicating that histone deacetylase inhibitor treatment is particularly efficient in inhibiting GBM cell invasion. Despite the absence of inhibitory effects of Apamin in 2D cell culture, G55T2 XTCCs revealed ∼70% reduced GBM invasion, associated with a substantial inhibition of proliferation as indicated by loss of Ki-67 positivity. Taken together, we show the suitability of the XTCC models for monitoring tumor growth and, in particular anti-invasive effects of drugs.

Glioblastoma (GBM) is a highly aggressive incurable brain cancer with limited treatment options (1, 2). Although current therapies can extend overall survival (2), prognosis is still poor, and a focus on identifying new therapeutic agents is imperative (1). Testing of novel drugs is an essential starting point for improving current therapies. However, reliable test systems that allow efficient testing of compounds in realistic tissue environments are needed to simulate patient-like conditions while maximizing screening efficiency. Most *in vitro* systems are straightforward to implement and facilitate high throughput efforts but fail to represent cellular interactions in the complex microenvironments of tumor masses and living tissues (3). This is particularly true for classical 2D cell cultures. On the other hand, *in vivo* models are highly realistic but labor-intensive, time-consuming, and require large numbers of animals, which also contradicts the 3R principle (4, 5). Tissue slice cultures may provide an efficient alternative by preserving the original tumor architecture *ex vivo* and requiring only 2 to 3 animals per experiment. While these systems are extensively used for culturing neuronal tissue (6, 7, 8), they have also been extended towards tissue slice models of primary tumors (9, 10, 11) and tumor xenografts (12). In the context of GBM, these tumor tissue slice models have been further extended toward so-called *ex vivo* tissue slice tandem co-culture models, more recently described by Sidorcenco and colleagues (13). While these systems were shown to also monitor GBM cell invasion into adjacent normal ‘host’ tissue, their applicability for assessing anti-glioma effects of established and promising drugs has not been studied. Since tumor cell invasion into surrounding normal brain tissue is a distinct issue in GBM patients and severely limits success rates of surgical intervention by only allowing partial resection, pharmacological interference in this process is of high relevance. Thus, the examination and monitoring of new promising drugs requires suitable models for precise analysis with, for example, classical Boyden chamber or migration assays being of only limited usefulness regarding the assessment of tumor cell-stroma interactions in an intact, viable 3D tissue environment.

So far, pharmacotherapeutic options are very limited in GBM. Temozolomide (TMZ) is a standard chemotherapeutic that is used in combination with radiation therapy, with limited efficacy by prolonging the overall survival of GBM patients for up to 14 months (14, 15). It causes DNA damage in GBM cells leading to reduced cell proliferation and tumor growth (16, 17). However, TMZ is not effective in approximately 50% of GBM patients (18) and can cause severe side effects (19). This limited success has prompted studies of other substances, such as the histone deacetylase (HDAC) inhibitor Vorinostat, as GBM therapeutics.

Vorinostat was originally approved for the treatment of cutaneous T-cell lymphoma (CTCL) (20). A few years later, the HDAC inhibitor was tested as monotherapy for recurrent GBM in a Phase II clinical trial (21), and in combination with TMZ and radiation therapy for newly diagnosed GBM in a Phase I/II clinical trial (22). Although Vorinostat has not become a standard therapy against GBM, some anti-tumorigenic effects could be observed, such as reduction of GBM cell migration and survival (23), alterations in GBM cell morphology (23), and increased radiosensitivity (22). Entinostat is a more selective, class I-specific HDAC inhibitor that has been shown to induce apoptotic effects and cell cycle arrest in lymphoma cells (24), reduce tumor growth, and improve antigen-presentation in ovarian cancer models (25). However, it has not been clinically tested yet in GBM.

In addition to newly developed or repurposed drugs tested in clinical trials, drugs from preclinical studies may be of potential interest as well. Apamin is a globular neurotoxic peptide consisting of 18 amino acids (26), that has been examined in preclinical studies and animal models. Isolated from bee venom, in which it constitutes 2 to 3% of the total dry mass (26, 27), Apamin can cross the blood-brain barrier (BBB) (28) and has been shown to selectively block the small-conductance calcium-activated potassium channels SK2 > SK3 > SK1 (29, 30, 31, 32). SK channels, which are activated by increased intracellular Ca^2+^, control K^+^ currents across plasma membranes and are ubiquitously expressed in the central nervous system (CNS) (32), skeletal muscle (33), and endocrine cells (34). Under physiological conditions, SK channels regulate neuronal firing frequencies (35) and cell volume (36), but in oncology, they have been linked to cancer cell migration (37, 38), invasion (39), and metastasis (39). In particular, the SK4 channel (K_Ca_3.1, a.k.a. intermediate-conductance channel, IK) was found to be upregulated in GBM tumor populations and GBM stem-like cells (GSC) (40) and is associated with increasing TMZ resistance (41), GSC motility (40), and GBM invasion (42). As treatment with Apamin reduced GBM cell line invasiveness in transwell assays (43), roles in GBM tumor biology might extend to the other channels of the SK family.

In this paper, we chose a panel of drugs either well-established or not yet extensively explored in GBM or other tumor entities, for testing in our XTCCs. The main focus was placed on monitoring the drug inhibitory effects of drugs on tumor cell penetration and infiltration into the normal adjacent brain tissue, as major issue in GBM.

## Results

### Tissue slice preparation, cultivation and analysis

**Figure 1:**
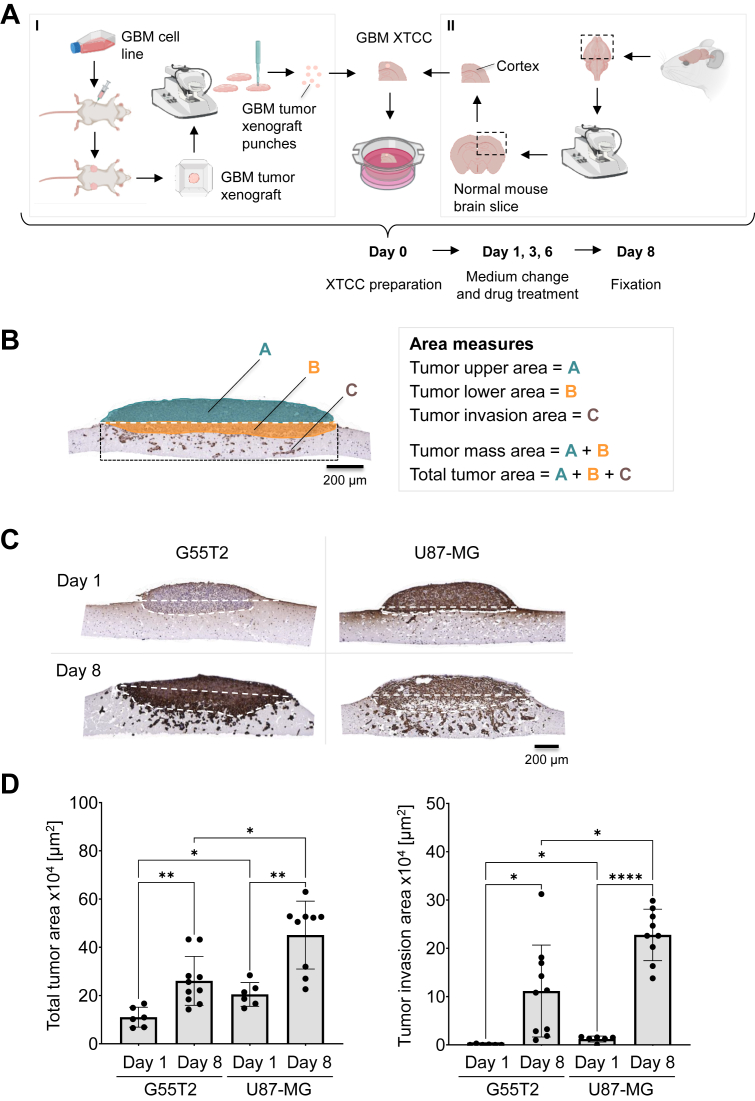
**Tissue slice preparation, cultivation, and analysis.***A*, preparation scheme of xenograft tissue slice tandem co-cultures (XTCC) consisting of the preparation of GBM tumor xenograft punches (I) and the preparation of normal cortical brain slices (II). The assembled XTCCs are cultured on air-liquid interface (ALI) transwell inserts over a period of 8 days. Medium change and drug treatment were applied on day 1, 3, and 6, followed by tissue fixation on day 8. Created with BioRender.com. *B*, example of the analysis and quantification of a Vimentin-stained XTCC. The measurement of the tumor upper area (*A*) and the tumor lower area (*B*) allows to determine the tumor mass area (A + B, *i*.*e*., space-assuming growth). The additional measurement of the tumor invasion area (*C*), consisting of the *brown-colored* single cells within the rectangle, enables to determine the total tumor area (A + B + C). *C*, representative microscopic pictures and (*D*) quantification of Vimentin-stained G55T2 and U87-MG XTCCs after a one- and 8-day cultivation period. Compiled data in swarm plots with histogram bars (mean ± SD) shows the total tumor area and tumor invasion area of cell line-based GBM xenografts after one and 8 days. Results are from at least two biological replicates with 6 to 10 technical replicates. Dunnett's T3 multiple comparisons test. Asterisks indicate statistically significant differences (∗, *p* < 0.05; ∗∗, *p* < 0.01; ∗∗∗, *p* < 0.001 and ∗∗∗∗, *p* < 0.0001). GBM, glioblastoma; XTCC, xenograft tissue slice tandem co-culture.

XTCCs were prepared as outlined in Figure 1*A* I + II. The subcutaneous injection of GBM cell suspensions into the flanks of immunodeficient mice led to the growth of tumor xenografts which, when reaching a size of about 10 mm, were excised and used for tissue slice preparation as described in Materials and Methods. Visibly necrotic areas or tumor parts with poor tissue preservation were discarded prior to sectioning. The 300 μm tissue sections were examined for intact tissue architecture and, using a biopsy stamp, punches of the tissue slice with 1.5 mm in diameter was prepared (Fig. 1*A* I). In a similar procedure, tissue slices were prepared from normal mouse brains containing the cortical region. (Fig. 1*A* II). After the normal brain tissue slices were laid onto the air-liquid interface (ALI) insert, the GBM tissue slice punch was firmly positioned on its top (day 0). The XTCCs were cultivated overnight, allowing the GBM xenograft tissue to attach to the normal brain slice prior to the first drug treatment on day 1 up to day 8. The microscopic evaluation of formalin-fixed, paraffin-embedded (FFPE) sections, vertically cut through both GBM layers and normal tissue allowed the evaluation of tumor cell penetration into the normal brain tissue. To distinguish GBM cells from the normal brain tissue, the FFPE sections were immunohistochemically stained with an anti-Vimentin antibody, leading to immunopositivity of the GBM tumor cells while leaving the normal murine brain tissue unstained. This enabled not only the differentiation of GBM and normal brain tissue but also, more importantly, the identification of even single invading GBM cells (Fig. 1*C*). Based on Vimentin-stained FFPE sections, two processes can be detected. First, the space-assuming growth of the bulk tumor mass into the normal brain tissue (Fig. 1*B*, ‘tumor lower area’ depicted in orange). Second, the ‘tumor invasion area’ which arises through parallel invasion of several single cells and/or their further cell division (Fig. 1*B*, depicted in brown), highlighting the characteristic properties of GBM tumors with regard to their invasive potential. To ensure a consistent analysis of the tumor invasion area (C) across the different XTCCs, which is independent of the size of the host tissue, only the brown-colored single cells below the tumor mass (within the rectangle) were included.

Notably, dependent on the cell line used for xenograft formation, differences in both processes were seen. While the tumor lower area of G55T2 cell-based xenografts profoundly increased over 8 days with comparably little single-cell invasion, the opposite was observed for U87-MG cell-derived xenografts (example given in Fig. 1*C*). In the latter case, the tumor lower area remained largely unchanged, while the tumor invasion area, representing the total area of invaded single cells, markedly increased. In both cases, however, tumor penetration into the normal brain tissue was substantial, with tumors reaching the lower end of the 300 μm brain tissue slice after an incubation period of only 8 days. ImageJ-based analysis (National Institutes of Health, imagej.org) of several sections from different XTCCs enabled a precise quantification. The total tumor area, comprising the bulk tumor mass (upper and lower tumor area) and invading tumor cells, was calculated as well. XTCCs derived from both cell lines showed total tumor growth over 8 days, indicating the viability of the co-cultures over this period. Notably, a particularly profound increase in the tumor invasion area over time was observed, indicating a substantial invasion of individual tumor cells into the normal brain tissue (Fig. 1*D*). As already indicated in Figure 1*C*, this is particularly true for U87-MG XTCCs (Fig. 1*D*, rightmost bars). Taken together, the observed growth and invasion patterns prove the suitability of the XTCC model for monitoring (space-assuming) tumor growth and, more importantly, tumor cell invasion.

### Assessment of inhibitory effects of Temozolomide in U87-MG and G55T2 xenograft tissue slice tandem co-cultures

**Figure 2:**
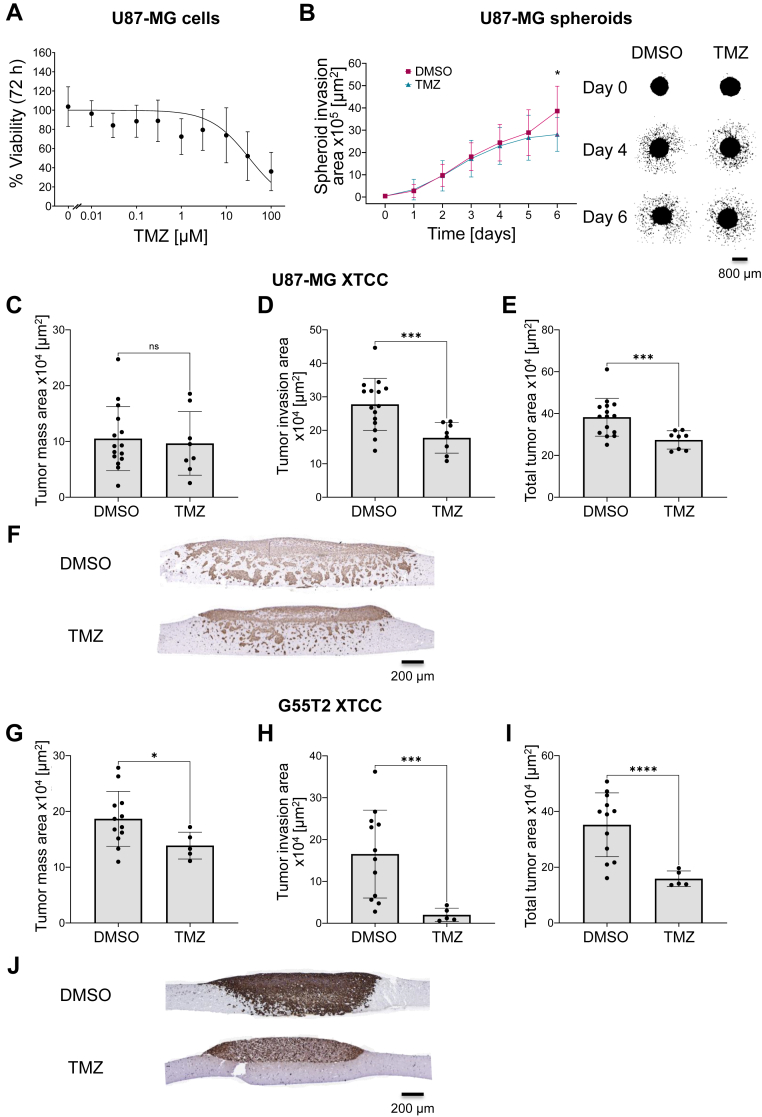
**Inhibitory effects of temozolomide (TMZ).***A*, TMZ dose-response curve (0.01–100 μM, mean ± SD) normalized to DMSO measured with WST-8 cell viability reagent using U87-MG cells. Results are from three biological replicates with 7 to 12 technical replicates. (*B*) Quantification (mean ± SD) of U87-MG spheroid invasion (*left*) and microscopic pictures (*right*) of U87-MG spheroids embedded in Hydrogel over 6 days of incubation. Results are from three biological replicates with 9 to 16 technical replicates. Unpaired *t* test with Welch's correction. (*C*–*E*) Swarm plots with histogram bars (mean ± SD) and (*F*) representative microscopic pictures of U87-MG XTCCs treated with 1% DMSO and 100 μM TMZ and immunohistochemically stained with Vimentin (8 days of total XTCC cultivation). *G*–*I*, Swarm plots with histogram bars (mean ± SD) and (*J*) representative microscopic pictures of G55T2 XTCCs treated with 1% DMSO and 100 μM TMZ and immunohistochemically stained with Vimentin (8 days of total XTCC cultivation). Results are from at least two biological replicates with 5 to 15 technical replicates. Unpaired *t* test with Welch's correction. *Asterisks* indicate statistically significant differences (∗, *p* < 0.05; ∗∗, *p* < 0.01; ∗∗∗, *p* < 0.001 and ∗∗∗∗, *p* < 0.0001).

To assess the capability of monitoring drug effects in our XTCC model, we first employed the well-established cytostatic drug Temozolomide (TMZ). For the selection of a suitable TMZ concentration, *in vitro* cell culture assays and the literature have been used. In a classical dose-response 2D cell culture assay with U87-MG cells, IC_50_ values of TMZ between 40 and 100 μM were observed (Fig. 2*A*). Additionally, a previous meta-analysis reported the common use of a median TMZ concentration of even above 100 μM in U87-MG cells (44). Thus, to ensure a sufficient matrix- and tissue-penetration of the drug, a TMZ concentration of 100 μM was selected for subsequent 3D and *ex vivo* experiments. As a classical 3D invasion assay, we first tested the outgrowth of GBM spheroids into a VitroGel Hydrogel Matrix solution. U87-MG spheroids invaded profoundly into the surrounding hydrogel matrix over a period of 6 days (Fig. 2*B*). When adding 100 μM TMZ to the matrix prior to spheroid embedding, inhibitory effects on spheroid invasion were only marginal (Fig. 2*B*, right). In stark contrast, when treating XTCCs with TMZ at the same concentration, profound invasion-inhibitory effects were observed. More specifically, in U87-MG XTCCs, the bulk tumor mass (upper and lower tumor area) remained largely unchanged (Fig. 2*C*), indicating no substantial toxic effects of 100 μM TMZ. However, a profound decrease in the tumor invasion area, *i*.*e*., the area representing the tumor cells individually invading the normal brain tissue, was observed (Fig. 2*D*). The total tumor area was reduced by about 15% over the 8-days incubation period (Fig. 2*E*). These differences in the invasion area between DMSO- and TMZ-treated XTCCs can also be visually observed in the representative microscopic pictures (Fig. 2*F*). Similarly, in G55T2 XTCCs, the bulk tumor mass was only marginally reduced upon TMZ treatment (Fig. 2*G*), while the tumor invasion area was significantly reduced by about 90%, indicating that the 100 μM TMZ treatment essentially abolished tumor cell invasion (Fig. 2*H*). The total tumor area was reduced by more than 50% (Fig. 2*I*). The similar bulk tumor masses and differences in the invasion area between DMSO- and TMZ-treated XTCCs can also be visually observed in the representative microscopic pictures (Fig. 2*J*). We conclude from these experiments that the XTCC model allows accurate monitoring of inhibitory effects on GBM growth and particularly on GBM invasion.

### TMZ-mediated inhibition of tumor cell proliferation (Ki-67) and apoptosis (Caspase-3) in G55T2 and U87-MG xenograft tissue slice mono-cultures and tandem co-cultures

**Figure 3:**
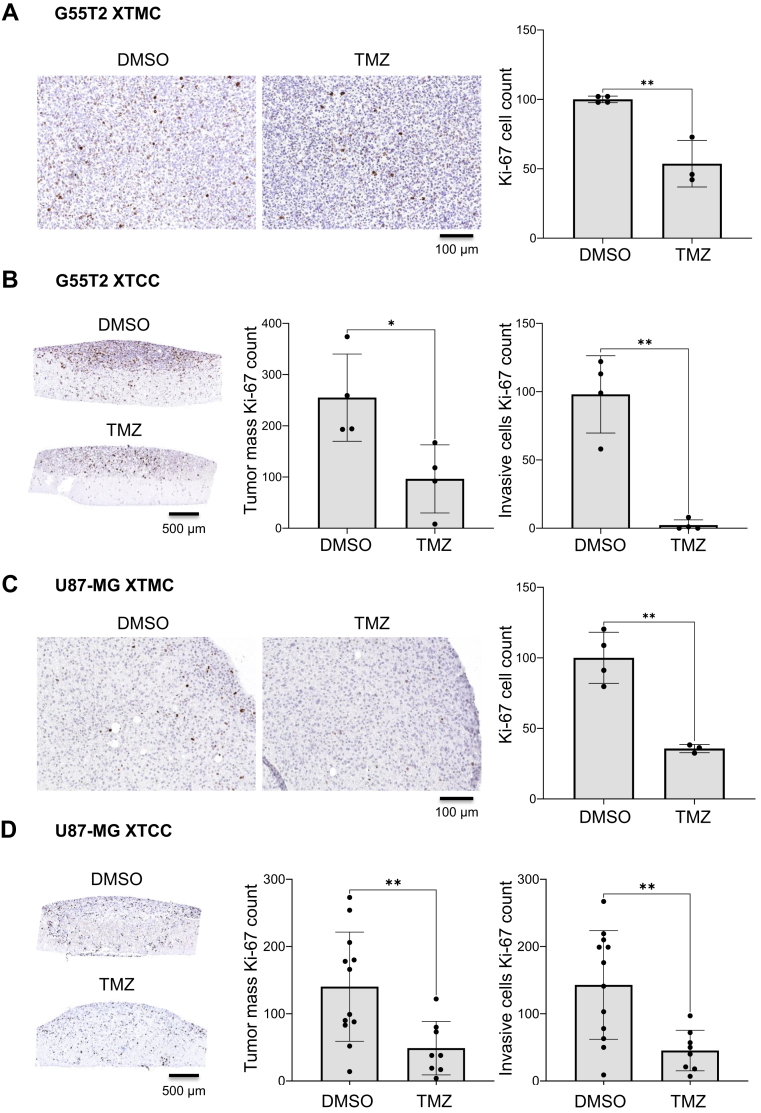
**Temozolomide-mediated inhibition of tumor cell proliferation.** Representative microscopic pictures and swarm plots with histogram bars (mean ± SD) of Ki-67-stained (*A*) G55T2 and (*C*) U87-MG xenograft tissue slice mono-cultures (XTMCs) treated with 1% DMSO and 100 μM TMZ (8 days of total XTCC cultivation). Results are from two biological replicates with 3 to 4 technical replicates. A standard unpaired *t* test was applied for statistical analysis. Representative microscopic pictures and swarm plots with histogram bars (mean ± SD) of Ki-67-stained (*B*) G55T2 and (*D*) U87-MG xenograft tissue slice tandem co-cultures (XTCCs) treated with 1% DMSO and 100 μM TMZ (8 days of total XTCC cultivation). Comparison of Ki-67 cell count within the tumor mass (upper and lower tumor area) and within invasive cells. Results are from at least two biological replicates with 4 to 12 technical replicates. An unpaired *t* test with Welch's correction was applied for statistical analysis. Asterisks indicate statistically significant differences (∗, *p* < 0.05; ∗∗, *p* < 0.01; ∗∗∗, *p* < 0.001 and ∗∗∗∗, *p* < 0.0001).

Anti-tumor effects upon drug treatment may rely on inhibition of tumor cell proliferation and/or induction of apoptosis. To further elucidate whether these effects can be monitored in our system, XTCCs were treated with 100 μM TMZ, and FFPE sections were stained for the proliferation marker Ki-67 and the apoptosis marker active Caspase-3. Microscopic pictures and quantification of Ki-67-stained G55T2 xenograft punches cultivated as xenograft tissue slice mono-culture (XTMC, *i*.*e*., without normal brain tissue underneath) and treated with TMZ revealed a 50% reduction of proliferating cells (Fig. 3*A*). In XTCCs treated with TMZ, this effect was also observed. Notably, the number of Ki-67-positive invading tumor cells was almost zero in XTCCs treated with TMZ, indicating that TMZ inhibited not only tumor cell invasion but essentially abolished their proliferation into the normal brain tissue (Fig. 3*B*). Profound proliferation inhibitory effects were also seen in U87-MG XTMCs (Fig. 3*C*) and XTCCs (Fig. 3*D*). In XTCCs, proliferation in the bulk tumor mass as well as in invading cells was reduced by more than 50% compared to the vehicle control.

For monitoring apoptosis induction upon TMZ treatment, FFPE sections were also stained for active Caspase-3 as a mediator of ex- and intrinsic apoptosis. No increase in active Caspase-3 was observed in G55T2 XTCCs upon TMZ treatment, while in U87-MG XTCCs a slight trend towards elevated Caspase-3 levels in TMZ treated XTCCs was seen. Overall, no substantial apoptosis was induced in co-cultures treated with 100 μM TMZ (Supporting Information Fig. S1, *A* and *B*).

### Inhibition of GBM cell invasion upon treatment of xenograft tissue slice tandem co-cultures with the HDAC inhibitors Vorinostat and Entinostat

**Figure 4:**
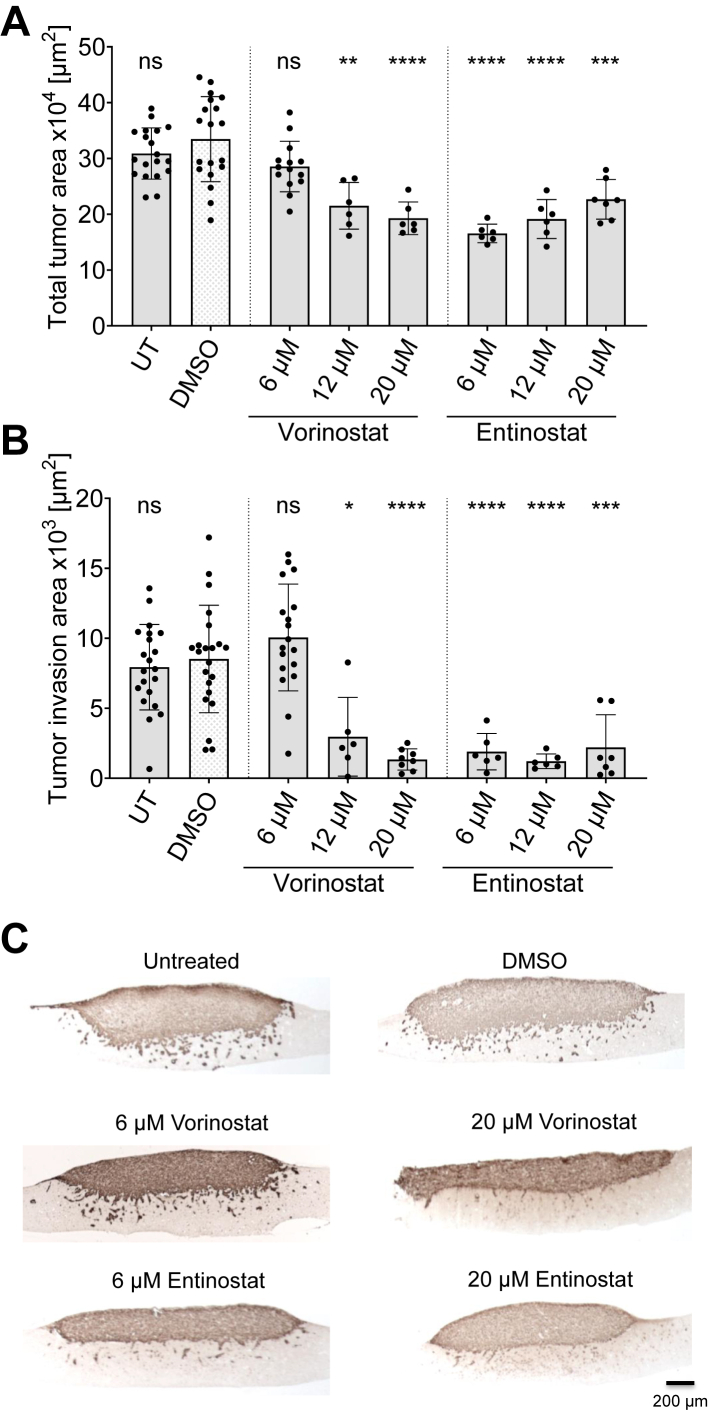
**Inhibitory effects of HDAC inhibitors vorinostat and entinostat.***A* and *B*, Swarm plots with histogram bars (mean ± SD) and (*C*) representative microscopic pictures of Vimentin-stained G55T2 xenograft tissue slice tandem cocultures (XTCCs) treated with 1% DMSO or different concentrations of Vorinostat and Entinostat (7 days of total XTCC cultivation). Results are from at least two biological replicates with 6 to 22 technical replicates. Unpaired *t* test with Welch's correction, ns = not significant. *Asterisks* indicate statistically significant differences (∗, *p* < 0.05; ∗∗, *p* < 0.01; ∗∗∗, *p* < 0.001 and ∗∗∗∗, *p* < 0.0001). XTCC, xenograft tissue slice tandem co-culture.

We further extended our studies towards two histone deacetylase (HDAC) inhibitors, Vorinostat and Entinostat, which have been examined in early-phase clinical trials in solid tumors (21, 45). Vorinostat is approved for the treatment of advanced, refractory cutaneous T-cell lymphoma (CTCL) and is explored alone and in combination with other drugs in different tumor entities (46, 47, 48, 49), including GBM (21, 22). Likewise, the selective class I HDAC inhibitor Entinostat was shown to induce cell cycle arrest and apoptosis in cells from different tumor entities (24, 50, 51) but has not been clinically tested for GBM. Based on published preclinical studies using concentrations of Vorinostat and Entinostat of about 1 to 10 μM (52, 53), G55T2 XTCCs were treated with Vorinostat and Entinostat at 6, 12, and 20 μM. Vimentin staining of FFPE sections containing XTCCs treated with Vorinostat revealed a slight reduction of the total tumor area, representing the bulk tumor mass as well as the invading cells (Fig. 4*A*). This effect was more profound in Entinostat-treated XTCCs, even though the total tumor area seems to rise slightly with increasing concentrations of Entinostat (Fig. 4*A*). This opposite dose-dependent effect might be due to changes in drug solubility or biological regulatory mechanisms that oppose external stimuli. Indeed, the analysis of G55T2 or U87 XTCCs upon Entinostat treatment revealed for example a substantial increase in the mRNA levels of the receptor tyrosine kinase HER3 (ErbB3), which forms heterodimers with other HER family members like HER2 and thus acts as a proto-oncogene (Supporting Information Fig. S2, *A* and *B*). Similar effects were found in the case of ligands of the EGF family. HB-EGF and TGFα, whose upregulation in various cancers has been shown to contribute to metastatic and invasive behavior and tumor progression, were chosen and revealed higher levels in XTCCs based on one or both cell lines (Supporting Information Fig.S2, *C*–*F*).

Notably, however, the tumor invasion area, *i*.*e*., the extent of single-cell invasion into the normal brain tissue, was found to be significantly inhibited by about 60 to 75% by both Vorinostat and Entinostat (Fig. 4*B*). This reduced cell invasion of Vorinostat- and Entinostat-treated XTCCs compared to untreated and DMSO-treated XTCCs can also be visually observed in the representative microscopic pictures (Fig. 4*C*). This indicates that HDAC inhibitor treatment is particularly efficient in inhibiting GBM cell invasion, with Entinostat showing higher efficacy than Vorinostat.

### Inhibition of GBM cell invasion upon treatment of xenograft tissue slice tandem co-cultures with the potassium channel blocker Apamin

**Figure 5:**
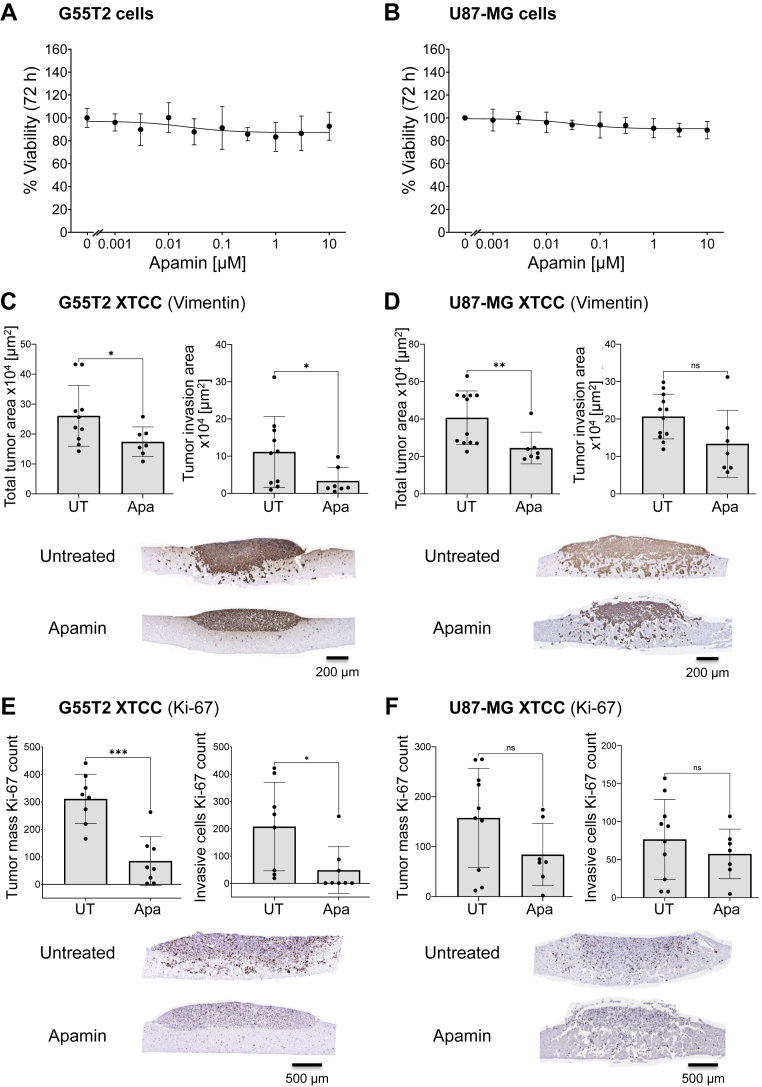
**Inhibitory effects of apamin.** Apamin dose-response curve (0.001–10 μM, mean ± SD) of (*A*) G55T2 and (*B*) U87-MG cell lines measured with WST-8 cell viability reagent. Results are from two biological replicates with 4 to 5 technical replicates. Swarm plots with histogram bars (mean ± SD) and representative microscopic pictures of Vimentin-stained (*C*) G55T2 and (*D*) U87-MG xenograft tissue slice tandem co-cultures (XTCCs) treated with 10 μM Apamin (8 days of total XTCC cultivation). Swarm plots with histogram bars (mean ± SD) and representative microscopic pictures of Ki-67-stained (*E*) G55T2 and (*F*) U87-MG XTCCs treated with 10 μM Apamin (8 days of total XTCC cultivation). Results are from at least two biological replicates with 7 to 12 technical replicates. Unpaired *t* test with Welch's correction, ns = not significant. *Asterisks* indicate statistically significant differences (∗, *p* < 0.05; ∗∗, *p* < 0.01; ∗∗∗, *p* < 0.001 and ∗∗∗∗, *p* < 0.0001). XTCC, xenograft tissue slice tandem co-culture.

The globular neurotoxic peptide Apamin is a selective blocker of the small-conductance calcium-activated potassium channels (SK2 > SK3 > SK1). The role of SK channels in cancer cell migration (37, 38), invasion (39, 43), and metastasis (39) prompted us to study possible Apamin effects in our XTCC model. Notably, classical 2D cell culture revealed no inhibitory effects on G55T2 or U87-MG cells (Fig. 5, *A* and *B*). In stark contrast, however, profound inhibitory effects of 10 μM Apamin treatment were seen in XTCCs. In G55T2 XTCCs, Apamin reduced again, particularly the tumor invasion area, by about 70% compared to untreated (UT) XTCCs (Fig. 5*C*). Inhibitory effects on the total tumor area were also seen in U87-MG XTCCs, however to a much lesser extent regarding the tumor invasion area compared to G55T2 XTCCs (Fig. 5*D*). Interestingly, the closer analysis of proliferation (Ki-67) revealed even more distinct differences between the cell lines. In G55T2 XTCCs, a very substantial reduction of Ki-67 positivity was seen in the bulk tumor mass and in the invading tumor cells (Fig. 5*E*), while essentially no differences in proliferation were seen in their U87-MG xenograft counterparts (Fig. 5*F*). When analyzing active Caspase-3, again, no increased apoptosis was detected in G55T2 and U87-MG XTCCs (Supporting Information Fig. S1, *C* and *D*).

### Evaluation of potential cytotoxic drug effects on normal cortical brain tissue slices of mice

A major benefit of the XTCC model is the ability to identify the effects of drugs on various parameters, including toxic and unwanted potential side effects on normal brain tissue. This is crucial for evaluating the tumor specificity and safety of a given substance. For closer examinations, we treated normal cortical brain slices of mice as above with 100 μM TMZ, 10 μM Apamin, 20 μM Entinostat, 20 μM Vorinostat, or 1% DMSO as vehicle control and monitored potential drug effects on apoptosis (active Caspase-3) and microglia (Iba1), changes in astrocytes (GFAP) and potential neuronal damages (NeuN) in the tissues (Supporting Information Fig. S3). No increased apoptosis was detected in the normal cortical brain slices upon an 8-days drug treatment period compared to the DMSO control. Similarly, astrocytic (GFAP), neuronal (NeuN), and microglial (Iba1) structures were detected in all brain slices, with no alterations in the cellular expression of these markers across the different treatment groups. Independent of the treatment, the immunohistochemical staining patterns of astrocytes and microglia were found to be quite heterogeneous. However, this is in line with the literature demonstrating that astrocytes and microglia would show an altered phenotype after longer cultivation times of the adult tissue. In fact, multiple studies identified astrocytic alterations during culturing of adult organotypic brain slices (54, 55, 56, 57, 58, 59). In the Iba1 staining, resting ramified microglia as well as more active cell states appearing as rounded cells with fewer cellular extensions were observed. Since these patterns were also detected in the control brain slices (untreated and vehicle control), the observed phenotypes cannot be ascribed to the drug treatments. Thus, it could be concluded that the normal cortical brain integrity is not negatively affected by the highest drug doses used in the co-culture assays.

### Combination treatment of Temozolomide with Entinostat

**Figure 6:**
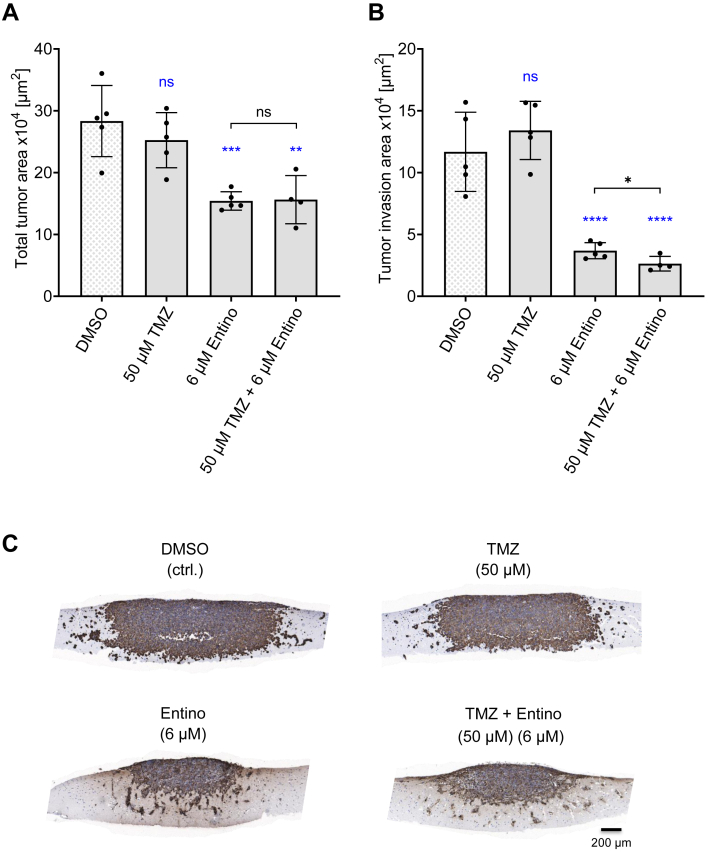
**Combination treatment of temozolomide (TMZ) with entinostat.** Swarm plots with histogram bars (mean ± SD) of (*A*) the total tumor area and (*B*) tumor invasion area, as well as (*C*) representative microscopic pictures of Vimentin-stained G55T2 xenograft tissue slice tandem co-cultures (XTCCs) treated with 1% DMSO or TMZ (50 μM) and Entinostat (6 μM), alone or in combination, for a total cultivation time of 8 days. Results are from one biological replicate with 4 to 5 technical replicates. *Black* symbols indicate pairwise comparisons using an unpaired *t* test with Welch's correction. *Blue* symbols indicate comparisons to the DMSO control using Dunnett's T3 multiple comparisons, ns = not significant. *Asterisks* indicate statistically significant differences (∗, *p* < 0.05; ∗∗, *p* < 0.01; ∗∗∗, *p* < 0.001 and ∗∗∗∗, *p* < 0.0001). XTCC, xenograft tissue slice tandem co-culture.

Since the HDAC inhibitor Entinostat substantially reduced G55T2 invasion in the XTCC model, we sought to analyze potential additive effects by combining Entinostat with TMZ (Fig. 6). Based on early preliminary experiments and the literature (60), a TMZ concentration of 50 μM was chosen and combined with a rather low 6 μM Entinostat dosage. G55T2 XTCCs were treated with TMZ and Entinostat, alone or in combination, for 8 days, followed by a Vimentin staining of the FFPE sections. At the selected dosage, TMZ alone did not reduce total tumor mass (Fig. 6*A*) or invasion (Fig. 6*B*) compared with the DMSO vehicle control, while 6 μM Entinostat showed profound inhibitory effects on both, as also evident in the representative microscopic pictures (Fig. 6*C*). The dual treatment with 50 μM TMZ + 6 μM Entinostat did not lead to a further decrease in total tumor area. However, despite the already ∼70% decrease in the tumor invasion area upon single Entinostat treatment, the combination of both drugs led to a slight, albeit statistically significant further reduction in tumor invasion. This validates the XTCC model also as a valuable tool for investigating and visualizing effects of drug combinations, with again distinguishing between bulk tumor growth and tumor invasion.

## Discussion

Reliable test systems ensuring a realistic study environment can be a crucial and time-saving step before new drug candidates enter *in vivo* studies and clinical trials. A promising *ex vivo* test system that creates a patient-like tissue environment and uses relatively few animals is the xenograft tissue slice tandem co-culture (XTCC) model. Details and advantages of this model for quantitative evaluation of growth and invasion have been described previously (13). Extending on that foundation, work here assessed the co-culture system as a model for testing pharmacological agents on GBM growth and, particularly, on invasion. Anti-cancer agents selected for the study included the established oral alkylating drug Temozolomide (TMZ) (61), two histone deacetylase (HDAC) inhibitors, Vorinostat and Entinostat, which have been examined in early-phase clinical trials (21, 45), and the neuroactive peptide Apamin, proposed as a blocker of invasiveness based on *in vitro* assays (43). Since inhibitory effects on cancer cell migration have been reported for all four drugs, they were chosen as test substances to evaluate the XTCC model as a suitable *ex vivo* test system.

TMZ is approved as a chemotherapeutic agent for the treatment of anaplastic astrocytoma and GBM (62, 63). It causes DNA damage and apoptosis through guanine methylation at the O6 position (16). Additionally, several studies have shown that TMZ alone or in combination with other drugs leads to cell cycle arrest, decreasing the proliferation marker Ki-67 (64, 65, 66, 67). Since the nuclear protein Ki-67 is highly expressed in all active phases of the cell cycle (G1, S, G2, M) but not in quiescent cells (G0), it is widely used as a marker for proliferative activity in cancer types, such as breast cancer (68, 69) and gliomas (70, 71). The detection of Ki-67 is also used as a classification factor to differentiate low- and high-grade gliomas (72, 73) as well as a prognostic factor correlating with the overall survival (OS) of isocitrate dehydrogenase (IDH) WT GBM patients (74). Since the anti-proliferative effects of TMZ are well-investigated, we monitored TMZ effects in our XTCC model as a starting point. Indeed, a reduction in GBM growth and invasion into the adjacent brain tissue could be observed upon TMZ treatment (Fig. 2, *F* and *J*). Interestingly, Ki-67 levels were not only reduced in the tumor mass but particularly in invasive cells (Fig. 3, *B* and *C*). Thus, besides the monitoring of drug effects on GBM tumor growth, the XTCC model allowed the detection of even more detailed drug effects and differentiation of the site of action.

To substantiate the drug effects that can be seen with the XTCC model, we tested two pharmacological agents, Vorinostat and Entinostat, that have already been examined in early-phase clinical trials in solid tumors. Both compounds were well tolerated and showed a modest activity (21, 45). Vorinostat has already been approved by the Food and Drug Administration for CTCL therapy (75), whereas Entinostat failed a Phase III clinical trial as adjuvant to an endocrine therapy in breast cancer patients due to lack of therapy improvement (76). Since Vorinostat has been clinically tested in GBM patients (21, 22) and Entinostat showed a reduction in tumor growth in preclinical studies of different cancer types (24, 50, 51), we sought to validate whether these effects could be seen in our GBM XTCC model, with a particular focus on GBM cell invasion. Both HDAC inhibitors showed a significant reduction in GBM tumor growth and an even more substantial inhibition of single-cell invasion at lower micromolar concentrations (Fig. 4). Especially Entinostat emerged as a promising drug candidate for GBM therapy, reducing cancer cell invasion to about 90% at only 6 μM. However, despite the strong anti-invasive effects and the ability of Entinostat to cross the BBB (77), it is important to note that only a low tissue penetration of Entinostat within the brain has been reported (78). Thus, approaches for BBB-permeability testing and drug optimization, for example through structural modifications (79), are crucial to enable an efficient GBM therapy. Although our XTCC model allows for the monitoring of drug effects in a complex tissue environment, the unfeasibility of identifying the BBB permeability of drugs is a clear limitation. Nonetheless, it is a valuable tool for determining a preselection of promising drugs, especially if an efficient BBB penetration is already confirmed, as it is the case for the globular peptide Apamin (28).

Apamin is a known inhibitor of SK channels (31). Especially SK2, which is blocked by Apamin with the highest sensitivity (29), seems to play a crucial role in multiple cancer types. Recently, Romito and colleagues showed that SK2 protein inhibition or silencing of the SK2 gene KCNN2 reduced cell migration but increased chemoresistance in ovarian cancer (38). Additionally, SK2 was reported to trigger pancreatic cancer tumorigenesis and enhance *in vitro* invasion and *in vivo* metastasis upon activation (39). Although SK2 is also expressed in GBM patient tissue (80, 81) and four SK2 isoforms have been found in U87-MG cells (82, 83), its exact function in GBM has not yet been clarified. However, new insights continue to emerge. A recent publication showed that the P01 toxin from scorpion venom decreased SK2 currents in U87-MG cells, resulting in reduced cell proliferation, adhesion, and migration (83). Furthermore, Varricchio and colleagues examined the effects of Apamin alone and in combination with an aquaporin 1 (AQP1) water channel inhibitor on the invasiveness of U87-MG and U251-MG cells (43). In U87-MG cells, Apamin reduced the invasion by about 55% and in U251 cells by about 20% (43). Combined treatment with the AQP1 water channel inhibitor AqB013 led to a further 20 to 40% reduction of invasion over Apamin alone (43), indicating key roles for ion channels such as SK2 in GBM tumor mechanisms, and supporting Apamin as a promising drug candidate in GBM therapy. Although clinical data on Apamin are still limited, it has several characteristics that render it a promising drug candidate for further studies, including its ability to cross the BBB (28), its neuroprotective features at lower micromolar concentrations (84), its anti-cancer (85, 86, 87) and anti-inflammatory (88, 89) effects, as well as its stability to serum proteases (28). Indeed, using the XTCC model, we could show that Apamin significantly reduces G55T2 xenograft invasion and restricts proliferating cells within the tumor mass, thereby preventing single-cell infiltration into the surrounding tissue (Fig. 5, *C* and *E*). Interestingly, the effects of Apamin on U87-MG xenograft invasion are lower compared to those on G55T2 xenografts, and its effects on U87-MG cell proliferation are also very marginal (Fig. 5, *D* and *F*). A reason for this could be the highly invasive nature of U87-MG cells (90, 91), which can also be distinctly observed in this model (Fig. 1*D*). Thus, the XTCC model not only allows for the identification of general anti-cancer effects but also displays more specific drug effects aligned with the characteristics of different GBM types.

Another notable advantage of the XTCC model is the ability to test compound toxicity on normal brain tissue and evaluate potential alterations in the structural organization of neurons and neuroinflammation by monitoring neuronal, microglial, and astrocytic parameters. This allows for the pre-selection of drugs based on their biocompatibility, which may lead to a reduction in the number of *in vivo* experiments.

Moreover, the XTCC model enables the facile testing of dual (Fig. 6) or multiple drug combinations independent of their potentially different pharmacokinetics, in order to identify potential additive or synergistic effects. According to previously reported TMZ/Entinostat combination treatment studies (60), a TMZ concentration of 50 μM was chosen and tested in combination with 6 μM Entinostat. While a single 50 μM TMZ dose was insufficient to reduce xenograft tumor growth or invasion compared with the DMSO vehicle control, this sub-pharmacological dosage led to a slight, but statistically significant enhancement of the Entinostat-mediated inhibition of tumor invasion. This demonstrates the potential of the XTCC model for investigating and visualizing effects of drug combinations.

Although the XTCC model exhibits several benefits over animal studies, such as the reduction of animal numbers as well as a simpler and more facile experimental procedure that also allows for simultaneously assessing more drugs, dosages and combinations, it also has some limitations compared to *in vivo* conditions. One significant disadvantage is the lack of an immune microenvironment and of a functional vascularization. To partially overcome these limitations, co-cultured spheroids consisting of different cell types (92, 93) or vascularized organoids (94) can be placed on the murine brain slices. While multiple cell-cell interactions and cytokine communications can be recreated in *ex vivo* models, the complete cellular diversity found in living organisms cannot be reproduced in 3D models. Additional factors to consider include the limited cultivation period of the co-cultures and potential differences in nutrient availability and oxygenation between living brain and tumor tissues. Since the XTCC model mainly relies on diffusion processes, drug effects may be underestimated in the absence of a functional vascular system or other active transport mechanisms beyond diffusion. Another important aspect is that several pharmacokinetic parameters cannot be addressed in the XTCC model. On one hand, this is a limitation, since *in vivo* studies are needed for comprehensive pharmacokinetic measurements, such as drug penetration through the BBB and systemic, in particular hepatic, metabolism of the test compounds. On the other hand, it can be of advantage for initially examining drug effects independently of drug pharmacokinetics, and thus before delving into more extensive studies on how the drugs are affected by their pharmacokinetic properties.

Additionally, it is well feasible to develop the XTCC model further towards higher clinical relevance, for example by using primary GBM cells or patient-derived xenograft tissue. The latter approach allows for the maintenance of the native tumor structure and cellular diversity (95, 96, 97). However, the establishment and cultivation of primary cells is labor-intensive, not easily reproducible and not readily scalable for high-throughput studies (95, 98). Another interesting approach for gaining further insight into the processes underlying tumor cell invasion in the context of the XTCC model may rely on live imaging by confocal microscopy for capturing invasion over time. Among others, this may provide quantifiable data on the speed, distance, and direction of invasive cells (99).

## Conclusion

The XTCC model is a powerful tool for monitoring drug effects on GBM growth and particularly on invasion, by generating a patient-like tissue environment and using relatively few animals. Although it cannot completely replace *in vivo* studies by, for example, not being able to simulate the intact BBB, the XTCC model is beneficial for identifying detailed drug effects on GBM tumors and normal brain integrity, as well as pre-selecting promising drugs for further studies.

## Experimental procedures

### Cell culture

The glioblastoma cell lines G55T2 and U87-MG were cultured at 37 °C at 5% CO_2_ and 96% humidity. G55T2 cells were generously provided by Dr Katrin Lamszus and cultivated in Iscove’s Modified Dulbecco’s Media (IMDM; Sigma-Aldrich) supplemented with 10% fetal bovine serum (FBS) and 2 mM L-alanyl-L-glutamine (Biochrom). U87-MG cells were obtained from the American type culture collection (ATCC). Cells were validated by STR profiling. Cell lines were cultivated in Dulbecco’s Modified Eagle’s Medium (DMEM; Sigma-Aldrich) supplemented with 10% FBS and 2 mM L-alanyl-L-glutamine and tested regularly for the absence of *mycoplasma* contamination. For subculturing, cells were washed briefly with phosphate-buffered saline (PBS), rinsed with trypsin-EDTA for 5 min at 37 °C, and resuspended in the corresponding cell culture medium with supplements. The cell number was determined with the Neubauer counting chamber.

### Pharmacological agents

The pharmacological agents used in this work comprise the oral alkylating drug Temozolomide (TMZ; Sigma Aldrich Co, St Louis, USA), the two HDAC inhibitors Vorinostat and Entinostat (both from MedChemExpress, Monmouth Junction, NJ, USA), and the neurotoxic peptide Apamin (Apa; Sigma-Aldrich Chemie). TMZ, Vorinostat, and Entinostat were solubilized in dimethyl sulfoxide (DMSO) to obtain 10 mM stocks, which were further diluted in the respective culture medium to attain the final concentrations used in the respective assays. The same quantity of DMSO (1%) served as vehicle control for these treatments. Apamin was solubilized in water to obtain a 500 μM stock, which was further diluted in the respective culture medium to attain the final concentrations used in the respective assays. Untreated samples served as control.

### Cell viability assay

Viable G55T2 and U87-MG cells were detected after 72 h drug incubation using the Cell Counting Kit-8 (Sigma-Aldrich, Darmstadt, Germany). Briefly, 1500 cells/well were seeded in a 96-well plate and cultured for 24 h. The respective drugs (TMZ: 0.01–100 μM, Apamin: 0.001–10 μM) were added to the cells and incubated for 72 h. The cell culture medium was removed from the plate. The colorimetric WST-8 reagent was diluted 1:10 with the respective cell culture medium, and 50 μl/well was added to the cells. After 1 h incubation at 37 °C, the plate was placed into the Multiskan FC Microplate Photometer (Thermo Fisher Scientific, Germany) and measured at a wavelength of 450 nm.

### Spheroid invasion assay

U87-MG cells were seeded in a round-bottom 96-well plate (25,000 cells/well) and incubated for 24 h at 37 °C for spheroid formation. The VitroGel Hydrogel Matrix solution (TheWell Bioscience) was mixed 2:1 (v/v) with cell culture medium (DMEM + 30% FBS) containing the respective drugs (DMSO, TMZ) in 3-fold concentration. The supernatant was removed from the spheroid plate and the hydrogel mixture was carefully added. After 20 min incubation at room temperature, the set hydrogel was covered with cell culture medium (DMEM + 10% FBS) containing the respective drugs in 1-fold concentration and incubated at 37 °C to complete the polymerization process. Pictures were taken every 6 h for 6 days using the Incucyte SX5 Live-Cell Analysis System (Sartorius).

### Generation of tumor xenografts, cortical brain slices, xenograft tissue slice tandem co-cultures (XTCC), and xenograft tissue slice mono-cultures (XTMC)

Immunodeficient NSG (NOD scid gamma) mice and athymic nude mice (Crl:NU(NCr)-Foxn^1nu^, Charles River Laboratories, Sulzfeld, Germany) were maintained at standard conditions and fostered by trained laboratory animal technicians, representing contemporary best practices, and allowed access to lab food and water ad libitum. All mouse studies were performed according to the national regulations of animal welfare and the national legislation on the use of animals for research (Animal Welfare Act, Animal Welfare Experimental Animal Ordinance) and were approved by the responsible research ethics committee and the local authorities (TVV36/18, TVV59/20 and TVV23/24, Saxony Regional Directorate, Leipzig). All animal experiments complied with the ARRIVE guidelines and were carried out in accordance with the EU Directive 2010/63/EU for animal experiments. This included measures to reduce animal suffering, the definition of humane endpoints (maximum tumor xenograft sizes) and euthanasia methods. The design and execution of the animal experiments were under strict and careful consideration of the 3R principles.

For the generation of GBM xenograft tumors (Fig. 1), G55T2 (2.5 x 10^6^ cells/150 μl) or U87-MG (5 x 10^6^ cells/150 μl) cells suspended in PBS were subcutaneously injected into both flanks of 3- to 6-month-old NSG or athymic nude mice. After two to 3 weeks, the formed xenograft tumors were isolated under aseptic conditions, embedded in warm 4% agarose, solidified on ice, and cut into 300 μm-thick slices using a Leica VT1200S Vibratome (Leica Microsystems). The tumor slices were kept on cold PBS.

6-well tissue culture (TC) plates were filled with either 1 ml/well RPMI 1640 cell culture medium (Roswell Park Memorial Institute 1640 Medium; Gibco, Thermo Fisher Scientific) supplemented with 10% FBS and 1% penicillin-streptomycin or with incubation medium as described in Sidorcenco *et al*. (13), and loaded with TC inserts for 6-well plates, (PET, transparent, pore size: 0.4 μm, Sarstedt). The plate was placed in a cell culture incubator at 37 °C and 5% CO_2_ to pre-warm the medium. Brain tissue slices were prepared from 3- to 4-month-old NSG mice. After surgical resection under aseptic conditions, the mouse brain was embedded in warm 4% agarose, solidified on ice, and cut into 300 μm-thick cortex slices using a Leica VT1200S Vibratome (Leica Microsystems, Nussloch, Germany). The slices were collected in cold PBS and immediately placed on the membrane of the trans-well inserts (Sarstedt) to create an ALI cultivation environment.

For the generation of a XTCC, the xenograft tumor slices were punched out using a 1.5 mm or 2 mm diameter biopsy stamp and placed on top of the 300 μm-thick cortex slices using two sterile spatulas. Punches for xenograft tissue slice mono-cultures (XTMC, *i*.*e*., without a tandem setting on a cortical brain slice) were prepared using a 3 mm diameter biopsy stamp and placed directly on the trans-well membrane. The plate was cultivated under standard conditions at 37 °C and 5% CO_2_ for 24 h to allow the tumor xenograft punch to attach to the cortical tissue before treatment.

### Cultivation and processing of normal brain slices, GBM xenograft tissue slice tandem co-cultures, and mono-cultures

On day 1, 24 h after the preparation of normal brain slices, co-cultures and mono-cultures, the culture medium was replaced with fresh medium containing the respective drugs or vehicle control (1% DMSO). The medium was replaced every other day. After a total incubation time of 7 to 8 days (always kept constant within each treatment series), the tissues were fixed with 4.5% formaldehyde (ROTIHistofix, Carl Roth) or with 4% paraformaldehyde (Merck Darmstadt, Germany) as described in Sidorcenco *et al*. (13) for 24 h and washed with PBS. The co-cultures were embedded in a warm matrix containing 2% agarose and 2.5% gelatine to physically protect the fragile constructs during tissue dehydration. The tissues were placed in tissue cassettes, stored in 70% ethanol for 24 h, and inserted in an automated tissue processor (Citadel 1000, Shandon/Thermo Fisher Scientific, UK) consisting of 70% ethanol (60 min), 80% ethanol (30 min), 96% ethanol (30 min), isopropanol (2 × 45 min), xylene (2 × 30 min), and paraffin (2 × 90 min; Pure Paraffin, MEDITE Medical). After dehydration, the tissues were embedded in paraffin (Tissue-Tek Embedding Console Model 4715), cut into 6 to 8 μm-thick slices (always kept constant within each treatment series) using a Leica Reichert-Jung Biocut 2035 microtome (Leica Biosystems), and collected on polysine adhesion glass slides (Epredia Polysine Adhesion Slides). To allow the tissue to dry and stretch, the slides were placed on a Leica Histoplate stretching table (Leica Biosystems) heated to 39 °C.

### Immunohistochemistry

Microscopic slides were incubated at 58 °C overnight. On the next day, the slides were heated in the microwave for 5 min at 700 W and incubated twice in Neo-Clear Xylene Substitute solution (Merck) for 10 min before being subjected to a decreasing alcohol series (100% EtOH, 96% EtOH, 70% EtOH). The slides were washed for 1 min in distilled water and 5 min in 1x PBS-Tween (PBS-T), followed by antigen retrieval at 85 to 95 °C in 1x sodium citrate buffer (pH 6, Vimentin staining) or 1x EDTA buffer (pH 8, Ki-67 staining) in the microwave for 15 min at 900 W. After 10 min cooling down at room temperature, the slides were washed again for 5 min in 1x PBS-T, followed by incubation in 0.3% hydrogen peroxide (H_2_O_2_) diluted in PBS-T for 30 min at 4 °C to block endogenous peroxidases. The slides were washed three times for 5 min in 1x PBS-T followed by application of the Liquid Barrier Marker (Pap Pen, Merck) around the tissue sections and blocking of non-specific binding sites in blocking solution (PBS-T + 2% BSA) with 10% Normal Horse Serum or 10% Normal Goat Serum (both from Vector Laboratories, Burlingame) for 1 h at room temperature. Finally, the slides were washed three times for 5 min in 1x PBS-T and incubated with the primary antibody (monoclonal mouse anti-Vimentin clone V9 (M0725), 1:1000, Dako Denmark A/S, Glostrup, Denmark; monoclonal mouse anti-human Ki-67 clone MIB-1 (M7240), 1:100, Dako Denmark A/S, Glostrup, Denmark; polyclonal rabbit active Caspase-3, 1:50, Enzo Life Sciences, monoclonal mouse anti-NeuN (neuronal nuclei), 1:100, Millipore; polyclonal rabbit anti-GFAP (glial fibrillary acidic protein), 1:1000, Dako Denmark A/S, Glostrup, Denmark, monoclonal rabbit anti-Iba1 (ionized calcium-binding adapter molecule 1), 1:1000, Abcam) diluted in blocking solution overnight at 4 °C. Control experiments were conducted without the primary antibody. On the next day, the slides were washed three times for 5 min in 1x PBS-T and incubated with the secondary antibody (biotinylated horse anti-mouse IgG or biotinylated goat anti-rabbit IgG, both from Vector Laboratories) diluted in blocking solution for 1 h at room temperature. Then, the slides were washed three times for 5 min in 1x PBS-T, incubated with avidin/biotin ABC complex reagent (Vectastain Elite ABC-HRP Kit, Vector Laboratories) diluted in PBS-T for 1 h at room temperature, and washed again three times for 5 min in 1x PBS-T. The staining solution (3,3′-diaminobenzidine tetrahydrochloride (0.1% DAB) with 0.003% H_2_O_2_ (both from Carl Roth)) was prepared and the slides were incubated in the staining solution until the brown staining could be observed under the microscope (Axioscope). The reaction was stopped by washing in distilled H_2_O, and nuclei were stained for 30 s with hematoxylin solution acc. to Gill II (Carl Roth). The slides with the stained slices were washed for 5 min with distilled H_2_O and dehydrated through an increasing alcohol series (70% EtOH, 96% EtOH, 100% EtOH). Finally, the slides were incubated for 5 min in n-butyl acetate (Carl Roth), mounted with mounting medium (Entellan, Merck), cover-slipped and allowed to dry overnight at room temperature. Microscopic pictures were taken with the All-in-One Fluorescence Microscope BZ-X810 (Keyence Corporation of America). Quantification of the Vimentin, Ki-67, and Caspase-3 staining was performed with the Image J software.

### RNA-Isolation and reverse transcription quantitative polymerase chain reaction (RT-qPCR)

1.0 × 10^5^ cells were seeded per well in a 6-well plate. Total RNA was extracted using the RNA Magic Reagent (Biobudget Technologies) following the manufacturer’s instructions. Reverse transcription was performed with the RevertAid RT Kit (Thermo Fisher Scientific). Subsequent quantitative PCR was carried out using the PerfeCTa SYBR Green FastMix ROX (QuantaBio) on a StepOnePlus Real-Time PCR System (Thermo Fisher Scientific). The master mix was prepared according to the manufacturer’s protocol, and the quantitative PCR was conducted under the following cycling conditions: initial activation at 95 °C for 2 min, followed by 45 cycles at 95 °C for 10 s, 55 °C for 15 s, and 72 °C for 15 s, with fluorescence measurements taken at the end of each cycle. For melting curve analysis, samples were incubated at 65 °C for 15 s and then heated up to 95 °C. Gene expression was normalized to RPLP0, with both RPLP0- and target-specific primers run in parallel for each sample. Relative expression levels were calculated using the formula 2ˆ(CP(actin) – CP(target)).

### Statistics

All statistical analysis and creation of diagrams were performed using the software GraphPad Prism Version 10.1.0 (graphpad.com). The data is presented as means ± sSD. Differences between three or more groups were determined by analysis of variance using one-way ANOVA. Differences between two groups were calculated either by a standard unpaired Student’s *t* test when the SDs and sample sizes of both groups were similar or with an unpaired Student’s *t* test with Welch’s correction when variances and sample sizes were unequal. Statistically significant differences are indicated as ∗, *p* < 0.05; ∗∗, *p* < 0.01; ∗∗∗, *p* < 0.001 and ∗∗∗∗, *p* < 0.0001.

## Data availability

We declare that all data supporting the findings of this study are available within the article and its supporting information.

## Supporting information

This article contains supporting information.

## Conflict of interests

The authors declare that they have no conflicts of interest with the contents of this article.
