# Supplementary material for: Xenograft tissue slice tandem co-cultures are a highly specific model to selectively analyze drug inhibitory effects on glioblastoma invasion
Source: J Biol Chem. 2025 Nov 25;302(1):110986. doi: 10.1016/j.jbc.2025.110986 (PMC12799928; doi:10.1016/j.jbc.2025.110986)
Supplement: Supplementary Material 1 [file mmc1.docx]

**Supplementary Figures Legend**

**Supporting Information Figure 1.** Swarm plots with histogram bars (mean ± SD) of active Caspase-3-stained (**A**) G55T2 and (**B**) U87-MG xenograft tissue slice tandem co-culture XTCCs treated with 1% DMSO or 100 μM TMZ (8 days of total XTCC cultivation). Swarm plots with histogram bars (mean ± SD) of active Caspase-3-stained (**C**) G55T2 and (**D**) U87‑MG XTCCs treated with 10 μM Apamin or normal cell culture medium (untreated, UT) as control (8 days of total XTCC cultivation). Results are from at least two biological replicates with 7 to 14 technical replicates. Unpaired t-test with Welch's correction, ns = not significant.

**Supporting Information Figure 2.** Swarm plots with histogram bars (mean ± SD) of mRNA levels of the proto-oncogenic receptor tyrosine kinase HER3 (**A, B**) and the ligands HB-EGF (**C, D**) or TGFα (**E, F**) in G55T2 and (**A, C, E**) or U87-MG (**B, D, F**) xenograft tissue slice tandem co-culture XTCCs treated with 1% DMSO or Entinostat at the indicated concentrations. Asterisks indicate statistically significant differences (*, p < 0.05; **, p < 0.01; ***, p < 0.001 and ****, p < 0.0001).

**Supporting Information Figure 3.** Representative microscopic pictures of cortical brain slices treated with the highest drug doses used in the co-culture assays (100 µM TMZ, 10 µM Apamin, 20 µM Vorinostat, 20 µM Entinostat, 1% DMSO) or untreated (UT). Sections of brain slices were stained with active Caspase-3 (arrows show positive cells), GFAP, NeuN, and Iba1.
