# Supplementary figures and images for: Xenograft tissue slice tandem co-cultures are a highly specific model to selectively analyze drug inhibitory effects on glioblastoma invasion
Source: J Biol Chem. 2025 Nov 25;302(1):110986. doi: 10.1016/j.jbc.2025.110986 (PMC12799928; doi:10.1016/j.jbc.2025.110986)

**A**

**G55T2 XTCC**

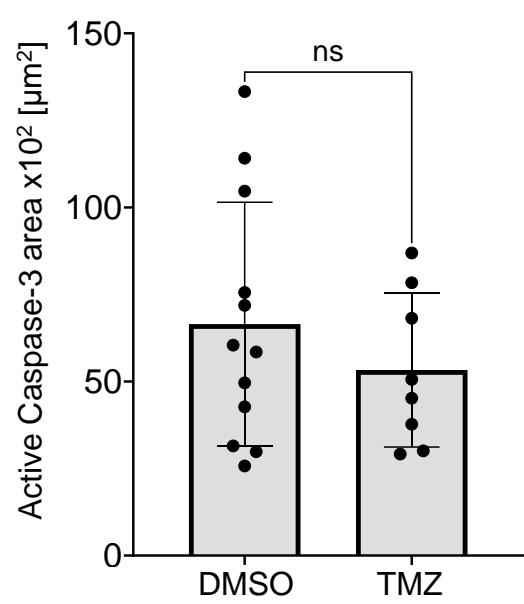

**B**

**U87-MG XTCC**

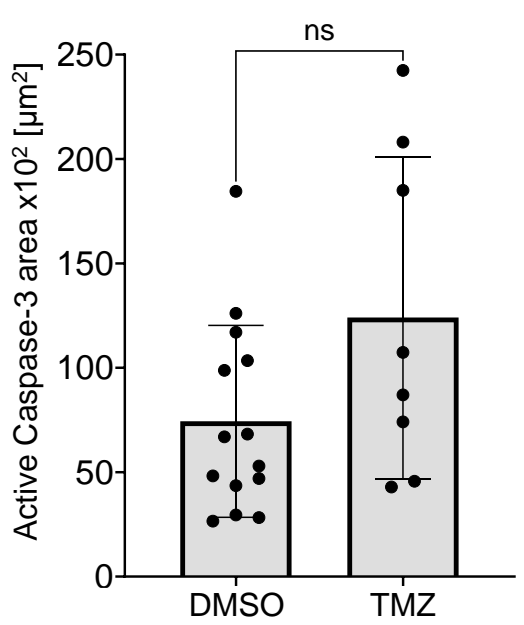

**C**

**G55T2 XTCC**

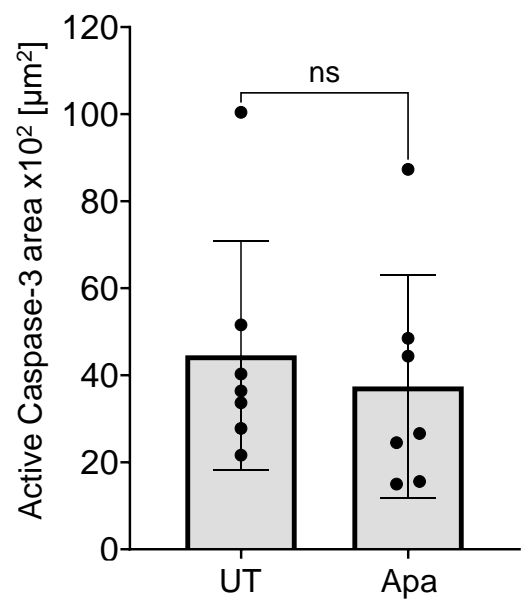

**D**

**U87-MG XTCC**

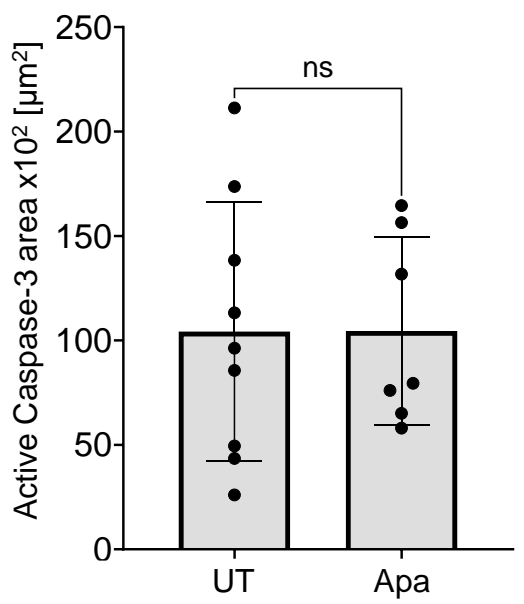

Supplement: Supplementary Material 2 [file mmc2.pdf]

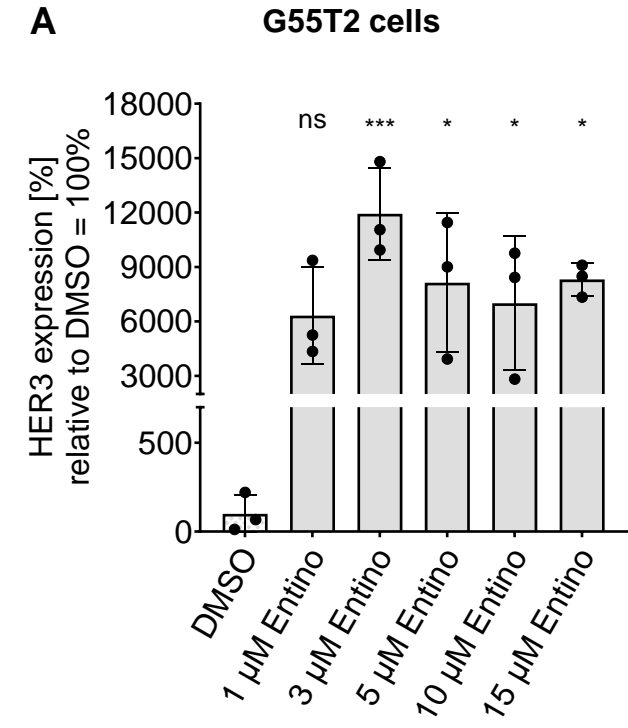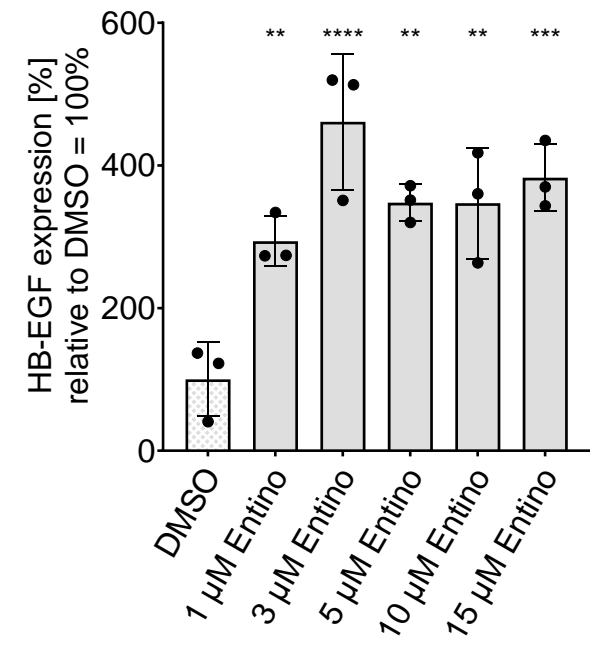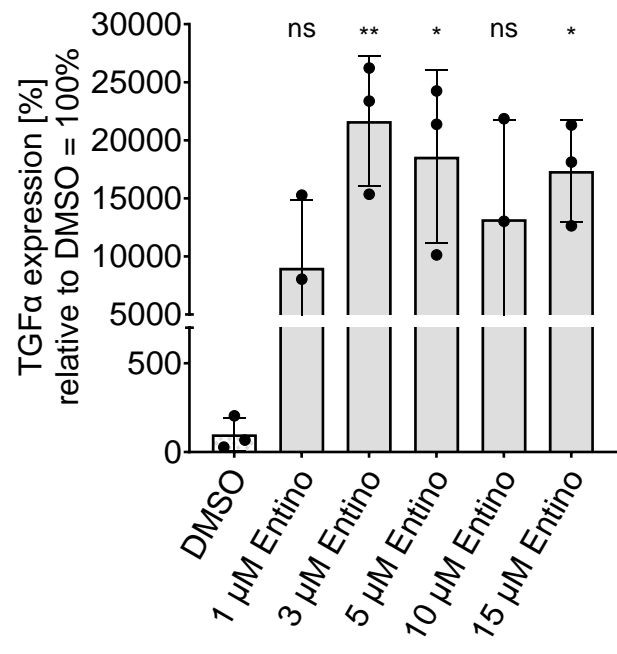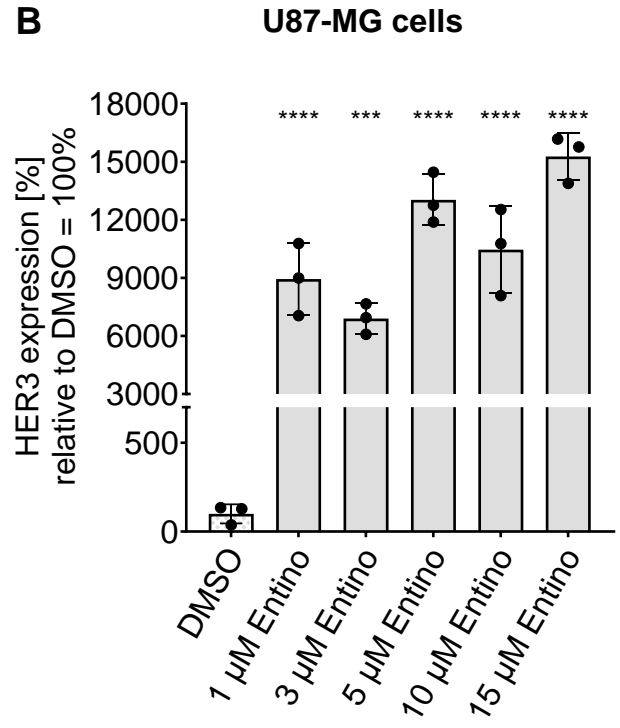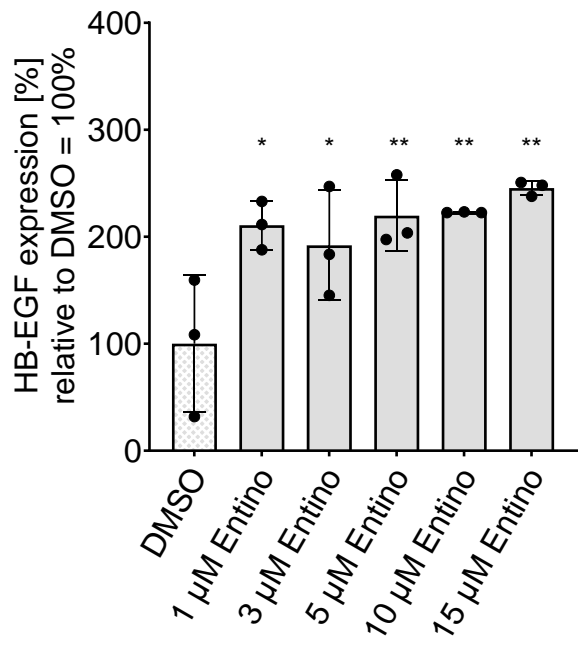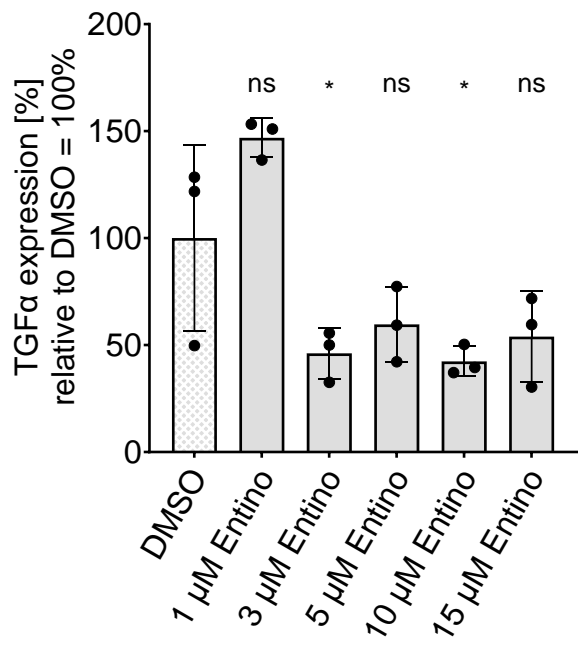

Supplement: Supplementary Material 3 [file mmc3.pdf]

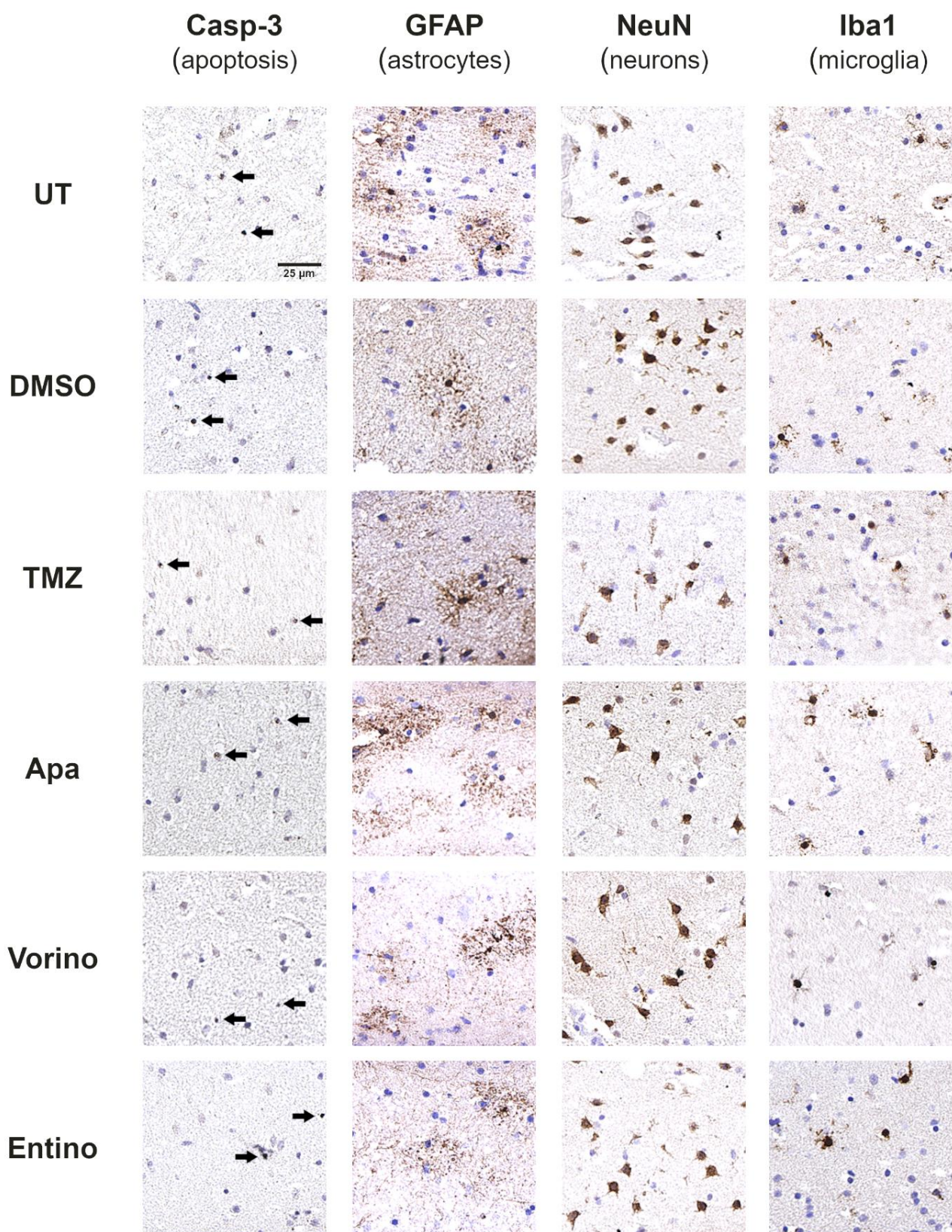

Supplement: Supplementary Material 4 [file mmc4.pdf]
